# Sporadic Erythromelalgia Associated with a Homozygous Carrier of Common Missense Polymorphism in SCN9A Gene Coding for NaV1.7 Voltage-gated Sodium Channel

**DOI:** 10.7759/cureus.4587

**Published:** 2019-05-02

**Authors:** Piotr K Janicki, Victor Ruiz-Velasco, Sanjib Adhikary

**Affiliations:** 1 Anesthesiology and Perioperative Medicine, Penn State Milton S. Hershey Medical Center, Hershey, USA

**Keywords:** erythromelalgia, pain management, single nucleotide polymorphism, nav1.7 sodium channels, whole exome sequencing

## Abstract

A 68-year-old female with a history of sporadic type and presumably secondary erythromelalgia with chronic intractable pain presented for foot surgery. The procedure was performed with combined general anesthesia and regional anesthesia consisting of the placement of a popliteal pain catheter for postoperative pain management. Subsequent whole-genome sequencing revealed that the patient was a homozygous carrier of the common missense mutation in the SCN9A gene coding for voltage-gated sodium channel (NaV1.7) - dbSNP rs6746030 (R1150W). The occurrence of this single nucleotide polymorphism (SNP) was previously suggested not to be associated with erythromelalgia but rather thought to be part of quantitative changes in the pain threshold in different cohorts of patients. The placement of the pain catheter, although controversial in patients with erythromelalgia, provided effective postoperative pain relief without any side effects.

## Introduction

Erythromelalgia is a disabling chronic pain condition characterized by chronic pain and erythema of the extremities, particularly feet, which is exacerbated by warmth and relieved by cooling. The erythromelalgia can be either secondary (mostly due to myelodysplasia), primary genetic (due to rare mutations in the SCN9A gene coding for NaV1.7 voltage-gated sodium channels), or sporadic (idiopathic), when no obvious etiology can be found [1-2]. We report here the identification of a homozygous carrier for the common missense single nucleotide polymorphism (dbSNP rs6746030) within the SCN9A gene in a chronic pain patient previously diagnosed with secondary erythromelalgia due to minimal myelodysplasia of the bone marrow and undergoing a surgical procedure for the lower extremities.

## Case presentation

The reported patient is a 68-year-old white female seen in the preoperative evaluation clinic for planned multiple toe amputations and tenotomies of the right foot due to osteomyelitis. The patient’s medical history included chronic pain syndrome as a consequence of secondary erythromelalgia. She also presented with a history of severe pain in her bilateral hands and feet, as well as a history of marked erythema of the palms of her hands and soles of her feet.

Other medical conditions included gastroesophageal reflux disease (GERD), chronic low back pain, previous atrial fibrillation episodes, and large and small fiber peripheral neuropathy of unknown etiology, causing occasional wrist drop. The chronic (several years) pain with more recent (three to four years) swelling and painful erythema of both feet prompted her to seek multiple medical consults several years ago. This led to bone marrow biopsy with a suspicion of erythromelalgia, which indicated mildly hypercellular bone marrow (70%) with maturing trilineage hematopoiesis, including minimal, normoblastic erythroid hyperplasia. The cytogenetic results, including fluorescence in situ hybridization assay, BCR/ABL1 gene sequence, and JAK gene V617F mutation studies, were all negative. The hematologic profile from peripheral blood indicated persistent anemia with an elevated red cell distribution width but a normal platelet number. The hematology consultant diagnosed the patient condition (in connection with clinical symptoms) as secondary erythromelalgia.

Because of the intractable chronic pain, the patient’s medication list included multiple medications associated with pain treatment consisting of oxycodone (40-60 mg daily), tramadol (200 mg daily), pregabalin (300 mg daily), nortriptyline (100 mg daily), aspirin (325 mg daily), ibuprofen 800 mg daily, and mexiletine (600 mg daily). In addition, the patient applied four daily patches of 5% topical lidocaine to the feet for 12 hours.

Due to the patient’s extensive history of intractable pain (including exacerbation of pain symptoms postoperatively with previous surgeries), we discussed the combined regional anesthesia, with general anesthesia as the anesthetic technique. For general anesthesia, the patient was preoxygenated and thereafter induced with propofol (150 mg) and ketamine (30 mg) administered intravenously. Intubation was facilitated with rocuronium and anesthesia was maintained with sevoflurane and a total of 150 mcg of fentanyl. No opioid narcotics or other analgesics were used intraoperatively. After 1.5 hours of an uneventful procedure, the patient was reversed, extubated, and transferred to the post-anesthesia care unit (PACU). Before emergence from anesthesia (secondary to the patient's refusal to place the block preoperatively), a peripheral nerve catheter (Pajunk E-Cath, Pajunk Medical Systems, Norcross, GA, US) was inserted perineurally at the right popliteal nerve using real-time ultrasound-guided visualization, followed by a bolus injection of 20 cc of 0.5% ropivacaine. An infusion of 0.2% ropivacaine was started after the initial bolus of local anesthetics. The infusion was delivered by an ambulatory infusion pump (Nimbus II PainPro, Infutronix Solutios LLC, Natick, MA, US) at 6 mL/hr. The patient required 15 mg PO oxycodone in the PACU and was discharged the same day with minimal pain (2/10 numerical pain score). The follow-up call the next day revealed that the patient was satisfied with her pain management (pain 5/10 on the numeric scale) and was instructed to continue infusion until the pump reservoir became empty. No additional complaints about worsening neurological status were obtained the next day or on a subsequent follow-up visit six weeks after the procedure.

Genotyping

Because of the patient’s history of erythromelalgia with an uncertain origin, we performed post hoc sequencing and genetic analysis of the total exome by next-generation sequencing. Blood deoxyribonucleic acid (DNA) was extracted, purified, and exome captured using the Agilent SureSelect Human All Exon V6 Kit (Agilent Technologies, CA, US). The whole exome sequencing strategy involved the creation of the 180-280 bp insert DNA library and sequencing on the HighSeq Illumina sequencer platform (Illumina, San Diego, CA, US) with an effective sequencing depth above 100×. The digital analysis pipeline of the raw data involved quality control and subsequent GATK-recommended strategy consisting of alignment sequence data (consisting of FASTQ files) with the reference genome (Hg19 and b37) using the GATK-Lite Variant Caller (Unified Genotyper from the GATK-like toolkit ver. 2.3; www.broadinstitute.org/gatk/gatkdocs/org_broadinstitute_sting_gatk_walkers_genotyper_UnifiedGenotyper.html), and run on DNAnexus platform (platform.dnanexus.com/). This application calls SNPs and/or indels in the input sequence files, which were further annotated with wANNOVAR (wannovar.wglab.org). In addition, the presence of complex variant and copy number variants of the exome was further examined using Freebayes variant caller and CVNator on the same DNAnexus platform [3-6]. The presence of two alleles for this SNP in the investigated DNA sample was further verified employing the real-time TaqMan real-time polymerase chain reaction (PCR) method using the commercial kit (C_29261054_10) from ThermoFisher Scientific (Waltham, MA, US), according to the manufacturer's instructions.

The results of the analysis focused on the exonic loci for the sodium-channel genes, especially the SCN9A gene, which are listed in Table 1. We identified 19 SNPs (nine nonsynonymous and 10 synonymous) in the loci containing voltage-gated sodium channel genes SCN1A - SCN11A, all present in the most current dbSNP database. We did not observe any of the previously reported mutations associated with erythromelalgia [7-9]. We noticed, however, that the patient was a homozygous carrier of missense and potentially damaging polymorphism rs6746030 in SCN9A, which was verified by the independent real-time TaqMan real-time PCR assay. All other SNPs in the investigated channels were either synonymous or were classified as benign.

**Table 1:** Common SNPs observed in the exonic region of SCN1A – SCN11A alpha-subunits of the voltage-gated sodium channel genes of the investigated patient by next-generation whole exome sequencing SNP: single nucleotide polymorphism

Chrom#	Alt allele	Ref allele	Gene	SNP type	Position of codon and protein change	dbSNP	Carrier status
3	T	C	SCN10A	nonsynonymous	NM_001293307:exon26:c.A4843G:p.M1615V	rs6599241	1/1
3	A	G	SCN10A	synonymous	NM_001293307:exon26:c.T4572C	rs6599242	1/1
3	A	G	SCN11A	synonymous	NM_014139:exon12:c.T1638C	rs4073113	1/1
2	C	T	SCN1A	nonsynonymous	NM_001165963:exon16:c.G3199A:p.A1067T	rs2298771	1/1
2	A	G	SCN1A	synonymous	NM_001165963:exon13:c.T2292C	rs6432860	1/1
2	T	C	SCN1A	synonymous	NM_001165963:exon9:c.A1212G	rs7580482	1/1
19	T	C	SCN1B	nonsynonymous	NM_199037:exon3:c.T629C:p.L210P	rs55742440	0/1
11	G	A	SCN3B	synonymous	NM_018400:exon3:c.C438T	rs1275085	0/1
17	T	C	SCN4A	synonymous	NM_000334:exon24:c.A4869G	rs2070720	0/1
17	T	C	SCN4A	nonsynonymous	NM_000334:exon23:c.A4126G:p.N1376D	rs2058194	0/1
17	T	C	SCN4A	nonsynonymous	NM_000334:exon10:c.A1570G:p.S524G	rs6504191	1/1
3	T	C	SCN5A	synonymous	NM_000335:exon17:c.A3183G	rs7430407	1/1
3	T	C	SCN5A	nonsynonymous	NM_000335:exon12:c.A1673G:p.H558R	rs1805124	0/1
3	T	C	SCN5A	synonymous	NM_000335:exon2:c.A87G	rs6599230	0/1
2	C	A	SCN7A	nonsynonymous	NM_002976:exon18:c.G2874T:p.M958I	rs6738031	0/1
2	G	T	SCN7A	nonsynonymous	NM_002976:exon2:c.C122A:p.T41N	rs7565062	0/1
2	A	G	SCN9A	nonsynonymous	NM_002977:exon19:c.T3448C:p.W1150R	rs6746030	1/1
2	A	G	SCN9A	synonymous	NM_002977:exon10:c.T1119C	rs13414203	0/1
2	C	T	SCN9A	synonymous	NM_002977:exon2:c.G174A	rs6432901	0/1

## Discussion

The two main forms of erythromelalgia are the primary and secondary types. Myeloproliferative diseases, together with thrombocythemia, are responsible for approximately 20% of cases of secondary erythromelalgia. The pathological signs in patients with secondary erythromelalgia include arteriolar intimal proliferation with thrombotic occlusions secondary to platelet aggregation [1-2]. Secondary erythromelalgia is most commonly associated with blood dyscrasias, connective tissue disorders, and drug reactions. Secondary erythromelalgia can result from a number of diseases such as myeloproliferative disorders (with a 3%-65% prevalence in patients with myeloproliferative disorders, especially polycythemia vera and essential thrombocytosis), hypercholesterolemia, autoimmune disorders, small fiber peripheral neuropathy, Fabry's disease, mercury poisoning, mushroom poisoning, sciatica, and some medications, including bromocriptine, verapamil, and ticlopidine [1-2].

Primary erythromelalgia (OMIM 133020), which is idiopathic or genetic, is a very rare autosomal dominant disorder. It represents channelopathy that is caused by rare mutations present in the SCN9A gene, which codes for the voltage-gated sodium channel NaV1.7 α subunit. These channels are preferentially expressed in the nociceptive dorsal root ganglion (DRG) [7-9]. Genomic studies in humans have provided compelling evidence emphasizing that high-impact rare mutations in hNaV1.7 with gain-of-function have marked effects on the channel function and clinical phenotype of familial primary erythromelalgia syndrome. The role of these mutations in pain pathways has been previously described [9]. The mutations associated with primary erythromelalgia are not always inherited as de novo mutations have been described.

**Figure 1:**
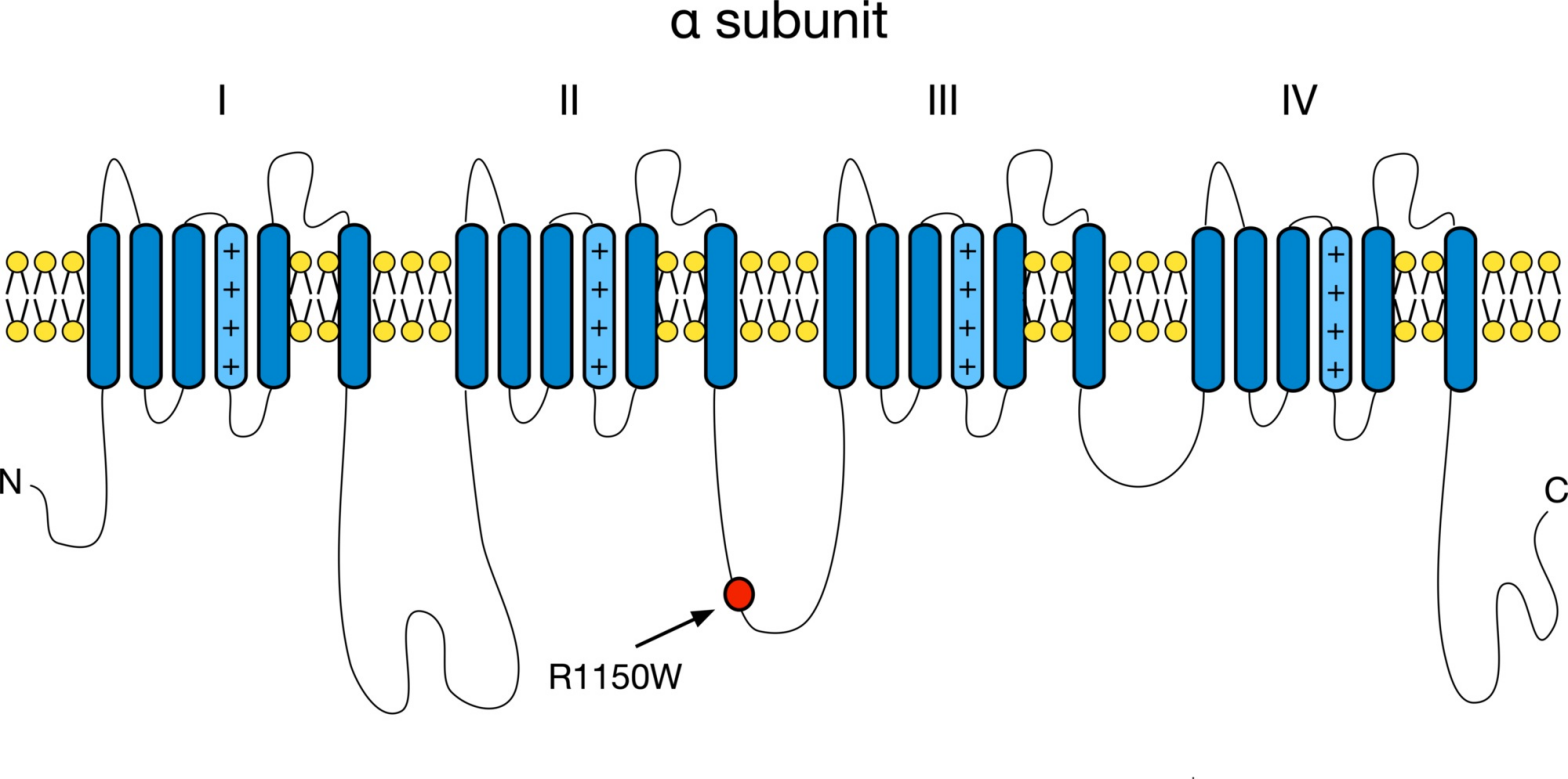
Nav1.7 alpha-subunit structure showing the location of amino acid affected by investigated common polymorphism rs6746030 is marked with the arrow

Common missense SNPs in the exons of the SCN9A gene have more subtle and quantitative effects than the major mutations mentioned above but may also significantly influence an individual’s pain perception. A common polymorphism (rs6746030) was previously found in the intracellular loop between domains II and III, with the minor allele occurring at a frequency of ~10%. The polymorphism results in a change of positively charged arginine (R, associated with minor A allele) to non-polar tryptophan (W, associated with the wild-type G allele) amino acid at residue 1150 in the reference human NaV1.7 sequence (Figure 1). The minor A allele was associated with increased pain perception when assessed in people with sciatica, amputees with phantom limb pain, and patients with post-discectomy lumbar root pain [10-17]. In addition, both clinical and experimental pain studies have shown that the level of pain felt was correlated with the genotype. That is, those patients with the least pain were wild-type GG genotype, intermediate pain in the GA genotype, and the most pain in the AA genotype, suggesting allelic dose dependency. To examine the functional effects of the rs6746030 SNPs, the two alleles that alter the coding sequence of NaV1.7, resulting in either 1150R (encoded by wild-type G allele) or 1150W (encoded by the minor A allele), were separately transfected into HEK-293 cells. The minor A allele was found to impair slow inactivation at voltages close to resting membrane potential, which is predicted to increase NaV1.7 activity [18]. In a different study, 1150W induced a depolarizing shift in the voltage dependence of activation compared with 1150R, inducing hyperexcitability in small DRG neurons, consistent with the clinical phenotype described in Reimann et al. [10-18]. These data suggest that SNPs in SCN9A resulting in NaV1.7 genetic variants may alter human pain threshold levels (i.e. produce quantitative changes in pain phenotype), and this variant may contribute to enhanced pain in common conditions such as back pain and osteoarthritis.

Furthermore, Drenth et al. (2005) reported the same variant in the heterozygotic carrier and diagnosis of sporadic erythromelalgia. However, it was proposed that because the variant does not affect a highly conserved nucleotide sequence, it does not cause the phenotype but is rather an innocent sequence variation [8]. The authors suggested that this variant may cause a pain phenotype with low penetrance (in contrast to most familial types of primary erythromelalgia mutations, which exhibit nearly 100% penetrance), or that its phenotype expression may be modulated by disease-modifier gene(s). It should be noted that Estacion et al. (2009) showed electrophysiological evidence that in rats, small DRG neurons transiently transfected with hNaV1.7 and that the polymorphism significantly increased the excitability of the neurons with the doubling of the firing frequency. In fact, the firing frequencies observed in DRG neurons transfected with the NaV1.7 1150W variant were similar to those seen in DRG neurons expressing the Q10R NaV1.7 mutant, which was found in a patient with primary erythromelalgia who had an onset of pain at 14 years, a greater age than typically observed in most patients with primary erythromelalgia [18]. It is unclear, in this respect, if the presence of two minor alleles of a frequent missense mutation in SCN9A - rs6746030 - could also be associated with intractable chronic pain symptoms in sporadic (as in the reported case) erythromelalgia. It is not clear why the patient with the Q10R mutation did not experience pain earlier and why most humans carrying 1150W NaV1.7 polymorphism do not develop chronic pain syndrome as primary erythromelalgia.

Because of the relative sparsity of homozygous rs6746030 carriers for the variant allele in the Caucasian population (3.29% in Coriell database samples - as reported by Estacion et al. (2009) - or less than 1% in our unpublished database), there are not many homozygous carriers of the variant allele to be investigated in cohort studies. Our observation suggests that the presence of two minor alleles in rs6746030 was related to intractable pain and erythromelalgia. In the majority of cases of secondary erythromelalgia reported so far, the chronic and tractable pain status is related to the degree of arteriolar inflammation, in which the platelet count is invariably significantly elevated. The patient in our report showed consistently normal levels of platelets. Similarly, this patient did not display signs of polycythemia vera and, in fact, the only one observed abnormality consisted of mild anemia and elevated red cells distribution width. It is also possible that our patient might represent the complex phenotype of mild erythromelalgia exacerbated by the presence of two variant alleles of the investigated polymorphism. Our observations in this study suggest the possibility that common missense polymorphism rs6746030 in NaV1.7 may not only contribute to the increased pain sensitivity or susceptibility to chronic pain but possibly, in at least some patients, contribute to symptoms of sporadic erythromelalgia.

The second interesting finding in this case report is the observed high efficiency of the peripheral continuous nerve block in managing the perioperative pain symptoms in the patient with erythromelalgia. Chronic pain symptoms in patients with erythromelalgia are very difficult to manage under conditions when a surgical procedure of the affected lower extremities is required. The evidence for or against performing peripheral nerve blocks in erythromelalgia is limited and not helpful because of concerns related to the exacerbation of underlying peripheral neuropathy [18]. Recently, Lorello et al. (2018) described the successful use of interscalene nerve block (single injection) as a complement to general anesthesia in a patient undergoing shoulder arthroplasty [19]. Thus, we reasoned that a similar approach of a continuous nerve block for the surgical procedure, along with a careful discussion of the benefits and risks with the patient, could be employed (for the first time reported in the literature for patients with erythromelalgia). Our results show that this approach led to the successful postoperative pain treatment with no neurological deficits. The six months follow-up (including neurological examination) did not find new neurological deficits or a worsening of erythromelalgia-associated pain status.

## Conclusions

Chronic pain associated with erythromelalgia may be associated not only with the severe myelodysplasia and carrier status for rare variants but also with the homozygous carrier status for common missense polymorphism rs6746030 R1150W. Continuous nerve block in the perioperative period represents a safe and efficient modality for postoperative pain management in erythromelalgia patients.
